# The role of lysosomal phospholipase A2 in the catabolism of bis(monoacylglycerol)phosphate and association with phospholipidosis

**DOI:** 10.1016/j.jlr.2024.100574

**Published:** 2024-06-09

**Authors:** Akira Abe, Vania Hinkovska-Galcheva, Philip Bouchev, Renee Bouley, James A. Shayman

**Affiliations:** 1 Department of Internal Medicine, University of Michigan, Ann Arbor, MI, USA; 2 Department of Chemistry and Biochemistry, The Ohio State University at Marion, Marion, OH, USA

**Keywords:** amiodarone, bis(monoacylglycerol)phosphate, cationic amphiphilic drug, lysosomal phospholipase, phospholipase A2 group 15, phospholipidosis

## Abstract

Bis(monoacylglycerol)phosphate (BMP) is an acidic glycerophospholipid localized to late endosomes and lysosomes. However, the metabolism of BMP is poorly understood. Because many drugs that cause phospholipidosis inhibit lysosomal phospholipase A2 (LPLA2, PLA2G15, LYPLA3) activity, we investigated whether this enzyme has a role in BMPcatabolism. The incubation of recombinant human LPLA2 (hLPLA2) and liposomes containing the naturally occurring BMP (*sn*-(2-oleoyl-3-hydroxy)-glycerol-1-phospho-*sn*-1’-(2′-oleoyl-3′-hydroxy)-glycerol (S,S-(2,2′,C_18:1_)-BMP) resulted in the deacylation of this BMP isomer. The deacylation rate was 70 times lower than that of dioleoyl phosphatidylglycerol (DOPG), an isomer and precursor of BMP. The release rates of oleic acid from DOPG and four BMP stereoisomers by LPLA2 differed. The rank order of the rates of hydrolysis were DOPG>S,S-(3,3′,C_18:1_)-BMP>R,S-(3,1′,C_18:1_)-BMP>R,R-(1,1′,C_18:1_)>S,S-(2,2′)-BMP. The cationic amphiphilic drug amiodarone (AMD) inhibited the deacylation of DOPG and BMP isomers by hLPLA2 in a concentration-dependent manner. Under these experimental conditions, the IC_50_s of amiodarone-induced inhibition of the four BMP isomers and DOPG were less than 20 μM and approximately 30 μM, respectively. BMP accumulation was observed in AMD-treated RAW 264.7 cells. The accumulated BMP was significantly reduced by exogenous treatment of cells with active recombinant hLPLA2 but not with diisopropylfluorophosphate-inactivated recombinant hLPLA2. Finally, a series of cationic amphiphilic drugs known to cause phospholipidosis were screened for inhibition of LPLA2 activity as measured by either the transacylation or fatty acid hydrolysis of BMP or phosphatidylcholine as substrates. Fifteen compounds demonstrated significant inhibition with IC_50_s ranging from 6.8 to 63.3 μM. These results indicate that LPLA2 degrades BMP isomers with different substrate specificities under acidic conditions and may be the key enzyme associated with BMP accumulation in drug-induced phospholipidosis.

Bis(monoacylglycerol)phosphate (BMP) is an acidic glycerophospholipid with a unique structure and stereochemistry (Fig. 1) (1). BMP is localized to late endosomal and lysosomal compartments and has been ascribed roles in many biological functions including the formation of internal vesicles in endosomes, the regulation of the trafficking and efflux of cholesterol in lysosomes in association with NPC1 and NPC2, the activation of lysosomal hydrolases such as acidic sphingomyelinase and β-glucocerebrosidase, the stabilization of endosome/lysosomal membranes via binding with Hsp70, and autophagosome-lysosome formation (2).

**Fig. 1:**
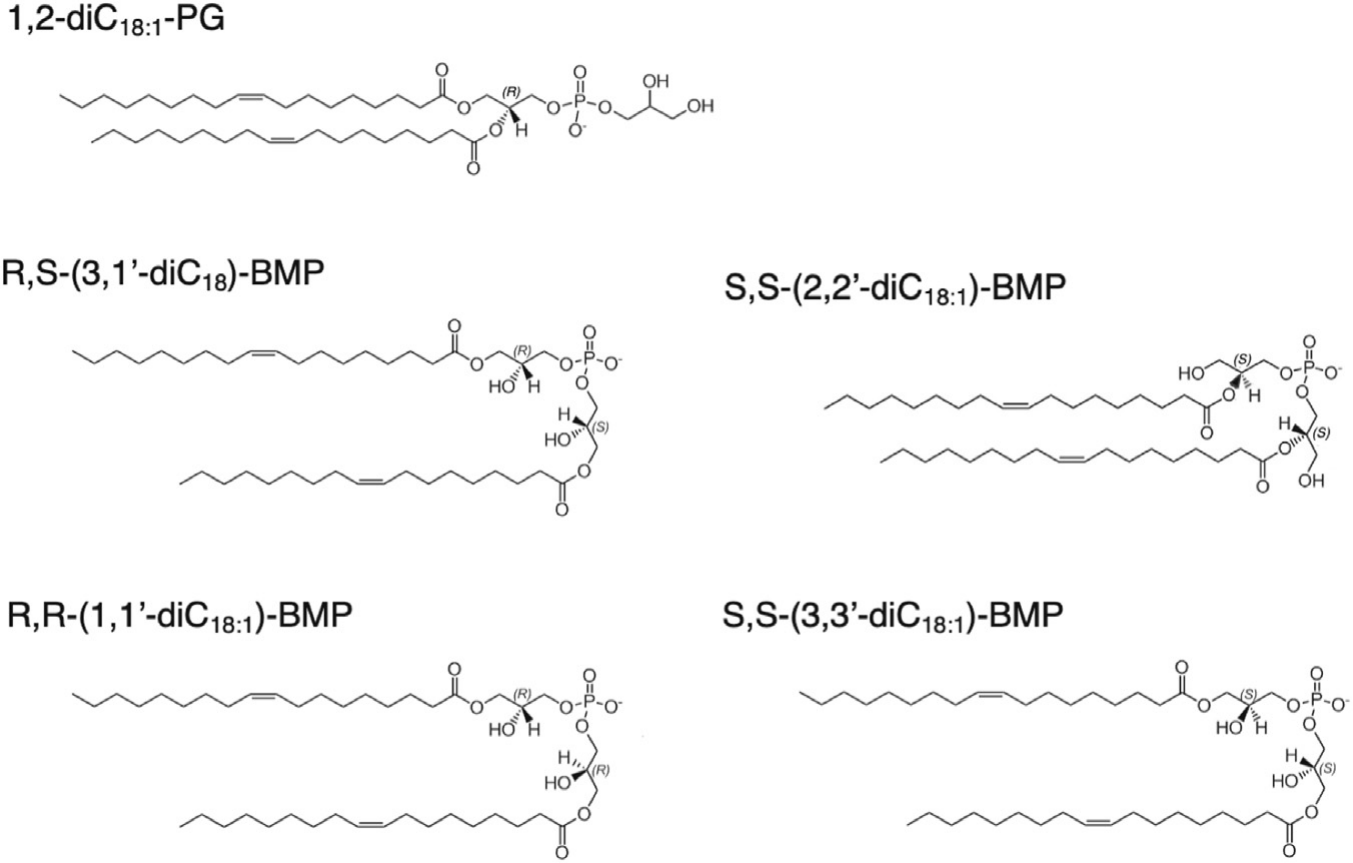
Structures of phosphatidylglycerol and the bis(monoacylglycerol)phosphate isomers used in this study.

Despite the growing evidence for the roles of BMP in these important biological processes, the metabolic pathways for BMP synthesis and degradation are not fully established. Phosphatidylglycerol (PG) is a likely precursor and its conversion to BMP requires de-acylation, transacylation, stereochemical rearrangement of R-configuration to S-configuration and finally re-acylation steps first proposed by Waite and colleagues (3, 4). Among each of these anabolic steps, the enzyme responsible for the re-arrangement of the *sn*-3:*sn*-1′ R-configuration to the *sn*-1:*sn*-1′ S-configuration is not known. Shinozaki and Waite suggested that a partially purified phosphatidylglycerol-selective lysosomal phospholipase A2 (PG-LPLA2) from RAW 264.7, macrophage-like cells, plays a crucial role in the first step involving a phospholipase A2 that cleaved the 2-acyl group from PG in the biosynthesis of BMPs (5). Interestingly, physicochemical properties and substrate and inhibitor specificities of the PG-LPLA2 activity by these authors were like those of the more recently characterized lysosomal phospholipase A2 (LPLA2), indicating that PG-LPLA2 might be identical to LPLA2. LPLA2 was initially identified by our group as a 1-*O*-acylceramide synthase due to its ability to transacylate short-chain ceramides and was subsequently identified as a phospholipase A2 with an acidic pH optimum. This enzyme is also referred to as group 15 phospholipase A2 (PLA2G15) and lysophospholipase 3 (LYPLA3).

Reported recently was that LPLA2 modulates BMP biosynthesis and cellular BMP levels in HeLa cells (6). However, this report is inconsistent with our past report of increased BMP levels in LPLA2 knockout mouse macrophages (7).

Phospholipidosis is an acquired lysosomal storage disorder resulting from exposure to a multitude of cationic amphiphilic drugs (CADs) (8). The cellular accumulation of BMP in the setting of (CAD)-induced phospholipidosis has been well established, and increased plasma BMP is used as a biomarker for exposure (9, 10). There have been no reports suggesting that CADs are activators of BMP synthesis. Therefore, BMP accumulation following CAD exposure may be the result of its impaired catabolism. Supporting this possibility is our earlier observations that alveolar macrophages of LPLA2-knock out mice express higher BMP levels than those of wild-type mice and that the increased content of BMP in the LPLA2 deficient macrophages was reduced by normal levels by exogenously added active LPLA2 (7).

Previous studies that followed our initial discovery and characterization of LPLA2 demonstrated that the activity of this phospholipase is inhibited by CADs as best exemplified by the anti-arrhythmic amiodarone (11). In this early study MDCK cells treated with CADs, amiodarone and the glucosylceramide synthase inhibitor PDMP, increase cellular phospholipid levels including BMPs (11). More recently, we have reported that the inhibition of LPLA2 activity is highly predictive of drug-induced phospholipidosis by scores of agents associated with lysosomal phospholipid accumulation in either in vitro or in vivo studies (12).

In the present study, we confirmed that LPLA2 degrades individual BMP isomers with different substrate specificities and that inhibition of LPLA2 activity against individual BMP isomers by amiodarone is more sensitive than that for phosphatidylglycerol. We also report that increased cellular BMP levels following treatment with amiodarone can be significantly reduced with the exogenous addition of active LPLA2 and that inhibition of BMP hydrolysis by LPLA2 results from exposure to a wide range of representative CADs.

## Materials and methods

### Reagents

**Supplemental Fig. 1:**
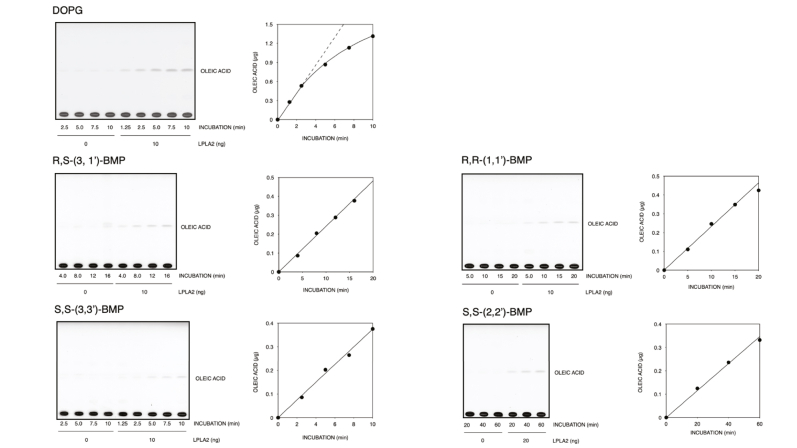
Initial deacylation curves for DOPG and BMP isomers. Ten ng of hLPLA2 was used in each reaction tube except for the S,S-(2,2′-diC18:1)-BMP assay, where 20 ng of LPLA2 was used. The net production of oleic acid by the enzyme (Y-axis) at each time point was determined by subtracting the oleic acid content in the absence of LPLA2 from the oleic acid content in the presence of LPLA2. For DOPG, the linear portion of the curve was observed between 0 and 2.5 min. The initial velocity of LPLA2 for DOPG were similarly determined. These were R,S-(3,1′- diC_18:_1)-BMP, 0–16 min; R,R-(1,1′-diC_18:1_)-BMP, 0–15 min; S,S-(3,3′-diC_18:1_)-BMP, 0–10 min; and S,S-(2,2′-diC_18:1_)-BMP, 0–60 min. Each panel represents one of three separate assays. The reaction curve in DOPG is the same as that shown in Fig. 2.

1,2-Dioleoylphosphatidylglycerol (DOPG), bis(monooleoylglycero)phosphate (BMP) isomers including *sn*-(3-(9Z-octadecenoyl)-2-hydroxy)-glycerol-1-phospho-*sn*-3’-(1’-(9Z-octadecenoyl)-2′-hydroxy)-glycerol (R,S-(3,1′,C_18:1_)-BMP), *sn*-(1-oleoyl-2-hydroxy)-glycero-3-phospho-*sn*-3’-(1′-oleoyl-2′-hydroxy)-glycerol (R,R-(1,1′,C_18:1_)-BMP) and *sn*-(3-oleoyl-2-hydroxy)-glycero-1-phospho-*sn*-1’-(3′-oleoyl-2′-hydroxy)-glycerol (S,S-(3,3′,C_18:1_)-BMP), 1,2-di-*O*-(9Z-octadecenyl)-*sn*-glycero-3-phosphocholine (DODPC), 1,2-dioleoylphosphatidylcholine (DOPC), sulfatide and *N*-acetylsphingosine (NAS) were obtained from Avanti Polar Lipids. *sn*-(2-oleoyl-3-hydroxy)-glycerol-1-phospho-*sn*-1'-(2′-oleoyl-3′-hydroxy)-glycerol (S,S-(2,2’,C_18:1_)-BMP) was from Echelon. The structures of DOPG and the four BMP isomers are shown in supplemental Fig. S1. Amiodarone, amlodipine, buclizine, carvedilol, chlorpromazine, chloroquine, cyclobenzaprine, lofepramine, loratadine, perhexiline, profenamine, propranolol, raloxifene, SB222200, seproxitine, tamoxifen, palmitic acid, oleic acid, tetrabutylammonium hydroxide solution (1M in methanol), and diisopropylfluorophosphate were from Sigma-Aldrich. Recombinant human LPLA2 was from Proteos, and high-performance thin layer chromatography (HPTLC) silica gel plates, 10 × 20 cm, were from Merck.

### Preparation of liposomes

Liposomes consisting of DODPC and PG or a BMP isomer in the molar ratio of 2.3:1, respectively, were used for the lipase reaction in which free fatty acid was released. Liposomes consisting of a BMP isomer, DODPC, and NAS in the molar ratio1:2.3:1.3 were used for the transacylase reaction in which 1-*O*-oleoyl-*N*-acetyl-sphingosine was formed. Each lipid dissolved in chloroform (C) or chloroform/methanol (C/M) (2:1, v/v) was delivered in a glass tube and dried down under a stream of nitrogen gas. The dried lipids containing DODPC and PG or BMP isomer were dispersed in 50 mM sodium acetate (pH 4.5) for 8 min by a probe-type sonicator while cooling in an ice-water bath. The resultant dispersions were mixed with bovine serum albumin (BSA) and adjusted to 10 μg/ml BSA.

### LPLA2 activity against PG and BMP isomers

The reaction solution consisted of 50 mM sodium acetate (pH 4.5), liposomes (100–130 μM of phospholipid: DODPC and PG or BMP isomer) and 10 μg/ml BSA. The reaction was initiated by the addition of LPLA2 to the reaction solution pre-incubated for 6 min at 37°C and kept for 1 to 20 min at 37°C. The reaction was terminated by adding C/M (2:1, v/v). The ratio of C/M/aqueous solution of the resultant mixture was 2:1:0.8, v/v. The organic phase obtained after the phase separation was transferred to a glass tube and dried under a stream of N_2_ gas. The dried reaction products were dissolved with C/M (2:1, v/v), applied to an HPTLC plate, and developed in a solvent system consisting of C/M/pyridine (98:1:2, v/v). After TLC, the plate is dried down by a hair drier and immersed in 8% (w/v) CuSO_4_•5H_2_O, 6.8% (v/v) H_3_PO_4_, and 32% (v/v) methanol. The uniformly wet plate was briefly dried by a hair dryer and charred for 15 min in a 150°C oven. The stained products were scanned by a CanoScan 9000F Mark II and analyzed by ImageJ. Known amounts of oleic acid were used to generate a standard curve.

### Inactivation of recombinant LPLA2

LPLA2 has a reactive catalytic serine residue that is inactivated by diisopropylfluorophosphate (DFP). Recombinant human LPLA2 (2–3 mg) was incubated with 20 mM DFP for 1 h at room temperature and dialyzed against 0.25 M sucrose, 10 mM Hepes (pH 7.4), and 1 mM EDTA at 4°C overnight. There was no difference in UV spectrum between untreated LPLA2 and DPF-treated LPLA2. DFP-treated LPLA2 completely lost enzymatic activity and was used as inactive LPLA2 in this study. LPLA2 activity assay was conducted using liposomes consisting of DOPC, sulfatide, and NAS as previously described (13).

### Amiodarone treatment of RAW 264.7 cells

RAW 264.7 macrophages were grown in 75 mm dishes in DMEM supplemented with nonessential amino acids and 10% fetal calf serum (FCS), 2 mM L-glutamine, 100 U/ml penicillin, and 100 mg/ml streptomycin (basal culture conditions) at 37 °C in a humidified atmosphere with 5% CO2. Cells were sub-cultured following trypsinization at a 1:5 ratio. RAW macrophages were seeded on day 0 into 35 mm diameter dishes (3 × 10^6^ cells per dish) in the medium containing FCS and incubated overnight. The medium was then replaced with FCS-free medium, and cells were treated with 0.312 μM amiodarone for 1, 3, 5, or 7 days in the absence of FCS. In additional dishes, the cells treated for 7 days with amiodarone were treated with the newly prepared medium containing 40 μg/ml hLPLA_2_ or 40 μg/ml DFP-inactive hLPLA_2_ for 24 h. Amiodarone-treated cells and LPLA2-or DFP-inactive LPLA2-treated cells were rinsed two times with PBS, scraped, and pelleted by centrifugation. Cell pellets were kept frozen at −80°C until analysis. The frozen pellets were resuspended in sucrose buffer and then sonicated by a probe-type sonicator while cooling in an ice-water bath (5 × 10 s). Protein content was determined by the bicinchoninic acid method. The cell homogenates were dispersed using C/M and the ratio of C/M/aqueous fraction was adjusted to 2:1:0.8 (v/v). The resultant organic phase was used to determine phospholipid and BMP contents. The total phosphate of lipid extracts was determined by the method of Ames (14). To determine BMP, lipid extracts were applied on an HPTLC plate, developed in a solvent system consisting of C/M/25%–28% ammonium hydroxide (65:35:5, v/v) and visualized as described in the LPLA2 activity assay above. μg.

### Statistical analysis

Data from three or more independent experiments were analyzed with a paired *t* test in GraphPad Prism 7 and expressed as mean ± SD. The differences between control and treated samples were considered statistically significant at *P* < 0.05.

## Results

### Degradation of S,S-(2,2′)-BMP by LPLA2

**Fig. 2:**
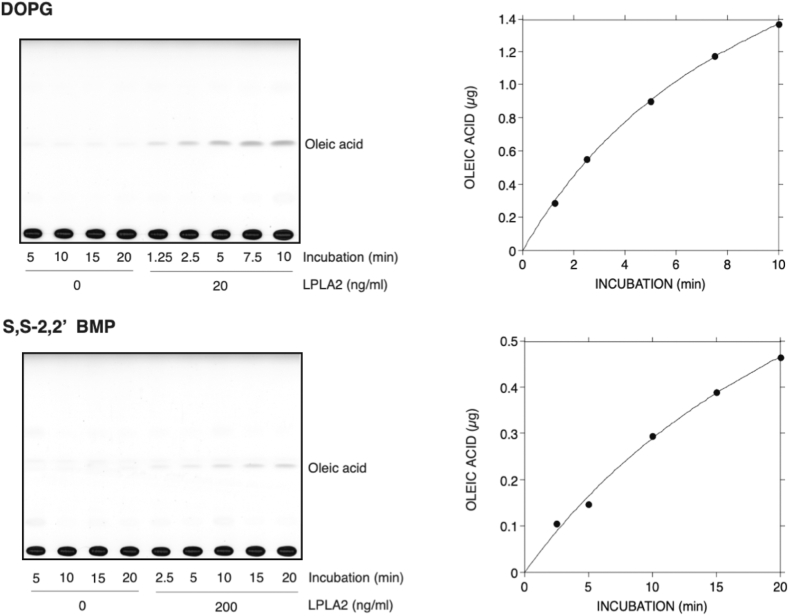
Degradation of 1,2-dioleoyl phosphatidylglycerol (DOPG) and *sn*-(2-oleoyl-3-hydroxy)-glycerol-1-phospho-*sn*-1'-(2′-oleoyl-3′-hydroxy)-glycerol (S,S-(2,2’,C_18:1_)-BMP) by LPLA2. De-acylation activity by LPLA2 was determined as described in Materials and Methods. The reaction mixture contained 70 μM DODPC and 30 μM DOPG or S,S-(2,2′,C_18:1_)-BMP as liposomes. The concentration of LPLA2 in the reaction mixture was 20 ng/ml and 200 ng/ml for DOPG assay and S,S-(2,2′,C_18:1_)-BMP assay, respectively. The left and right panels show TLC plates and their kinetic curves, respectively.

**Fig. 3:**
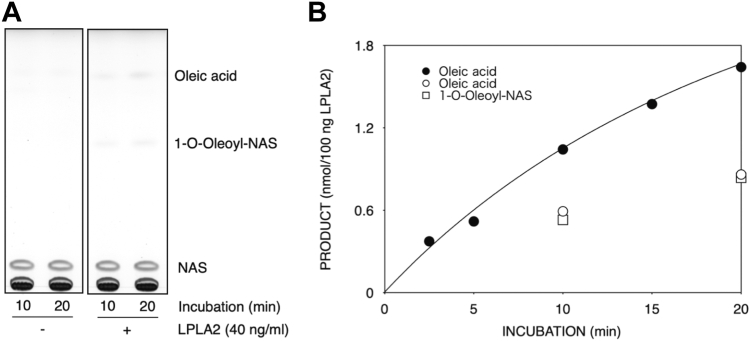
Degradation of (S,S-(2,2′,C_18:1_)-BMP) by LPLA2 in the presence of *N*-acetyl-sphingosine (NAS). Deacylation and transacylation activities by LPLA2 were determined as described in Materials and methods. The reaction mixture contained 70 μM DODPC, 30 μM S,S-(2,2′,C_18:1_)-BMP and 40 μM NAS as liposomes. The concentration of LPLA2 in the reaction mixture was 40 ng/ml. A: representative thin layer chromatogram demonstrating the formation of oleic acid and 1-*O*-oleoyl-N-acetylsphingosine in the presence of LPLA2. B: time dependent formation of oleic acid and 1-*O*-oleoyl-N-acetylsphingosine in the presence or absence of N-acetylesphingosine in the reaction mixture. Oleic acid and 1-*O*-oleoyl-NAS produced by LPLA2 in the presence of NAS were plotted by open circles (**○**) and open squares (**□**), respectively. The reaction rate curve indicated by closed circles (•) was same as that shown in the Fig. 1.

To determine whether LPLA2 can degrade the naturally occurring form of BMP, the de-acylation of S,S-(2,2′,C_18:1_)-BMP by LPLA2 was compared to that of DOPG in vitro. Liposomes consisting of DODPC/DOPG or DODPC/S,S-(2,2′,C_18:1_)-BMP were incubated with hLPLA2 at pH 4.5 (Fig. 2). DODPC is a 1,2-di-*O*-alkylglycerophospholipid that is not cleaved by LPLA2. The concentration of hLPLA2 in the DODPC/S,S-(2,2′,C_18:1_)-BMP liposomes assay was 10-fold higher than that in the DODPC/DOPG assay. Under these conditions, the release of oleic acid from S,S-(2,2′,C_18:1_)-BMP by LPLA2 was readily observed (Fig. 2). The specific activity of LPLA2 for S,S-(2,2′,C_18:1_)-BMP is estimated to be 60–70 times lower than that for DOPG from right panels in Fig. 2. In addition, when DODPC/S,S-(2,2′,C_18:1_)-BMP liposomes incorporated with NAS were used to compare phospholipase and acyltransferase activities, both 1-*O*-oleoyl-NAS formation and oleic acid release by LPLA2 were observed (Fig. 3). The initial rate of 1-*O*-oleoyl-NAS formation was comparable to that of oleic acid release and was half of that of oleic acid release observed in Fig. 1. The formation of 1-*O*-oleoyl-NAS indicates that S,S-(2,2′,C_18:1_)-BMP behaves as a substrate for LPLA2 like other glycerophospholipids in which this transacylase activity is observed.

### Substrate specificity

**Fig. 4:**
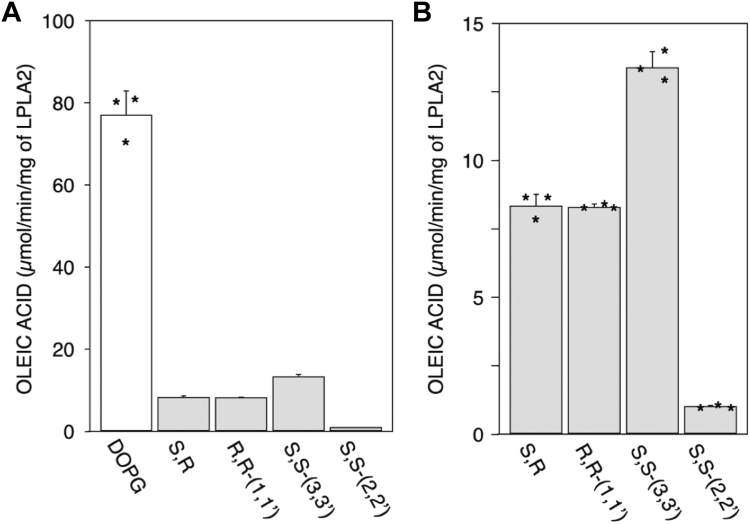
Substrate specificity. LPLA2 was incubated with liposomes consisting of 70 μM DODPC and 30 μM DOPG or 30 μMBMP isomer such as R,S-(3,1′,C_18:1_)-, R,R-(1,1′,C_18:1_)-, S,S-(3,3′,C_18:1_)- and S,S-(2,2′,C_18:1_)-BMPs. The specific activities of LPLA2 for DOPG and BMP isomers were determined by the initial velocity using a linear kinetic curve for each reaction (A) as shown in supplemental Fig. S1. Panel B shows an expanded alignment of the specific activity for BMP isomers shown in Panel A. Asterisks (∗) indicate the distribution of LPLA2 activity on each substrate.

Four commercially available BMP isomers, R,S-(3,1′,C_18:1_)-, R,R-(1,1′,C_18:1_)-, S,S-(2,2′,C_18:1_)- and S,S-(3,3′,C_18:1_)-BMPs, were next evaluated. To determine substrate specificity of LPLA2 to these lipids, DOPG and the individual BMP isomers were incorporated into DODPC based liposomes and incubated with LPLA2. The specific activities of LPLA2 for each isomer were obtained by measuring the initial velocity of oleic acid release with linear kinetics (supplemental Fig. S1). The specific activity of LPLA2 for DOPG was 75 times higher than that for S,S-(2,2′,C_18:1_)-BMP (Fig. 4A). In addition, R,S-(3,1′,C_18:1_)-, R,R-(1,1′,C_18:1_)- and S,S-(3,3′,C_18:1_)-BMPs were deacylated by LPLA2 at different rates (Fig. 4A, B), albeit still lower than the de-acylation rate for DOPG by LPLA2. The specific activity of LPLA2 for DOPG was 9.2, 9.3 and 5.8 times higher than those for R,S-(3,1′,C_18:1_)-BMP, R,R-(1,1′,C_18:1_)-BMP and S,S-(3,3′,C_18:1_)-BMP, respectively (Fig. 4B). The specific activity for S,S-(3,3′,C_18:1_)-BMP was higher than other BMP isomers. Despite having similar chirality, a 13-fold difference in substrate specificity was observed between S,S-(2,2′,C_18:1_)-BMP and S,S-(3,3′,C_18:1_)-BMP (Fig. 4B).

#### Substrate preference by LPLA2 for S,S-(2,2′, C_18:1_)-BMP versus S,S-(3,3′, C_18:1_)-BMP

**Fig. 5:**
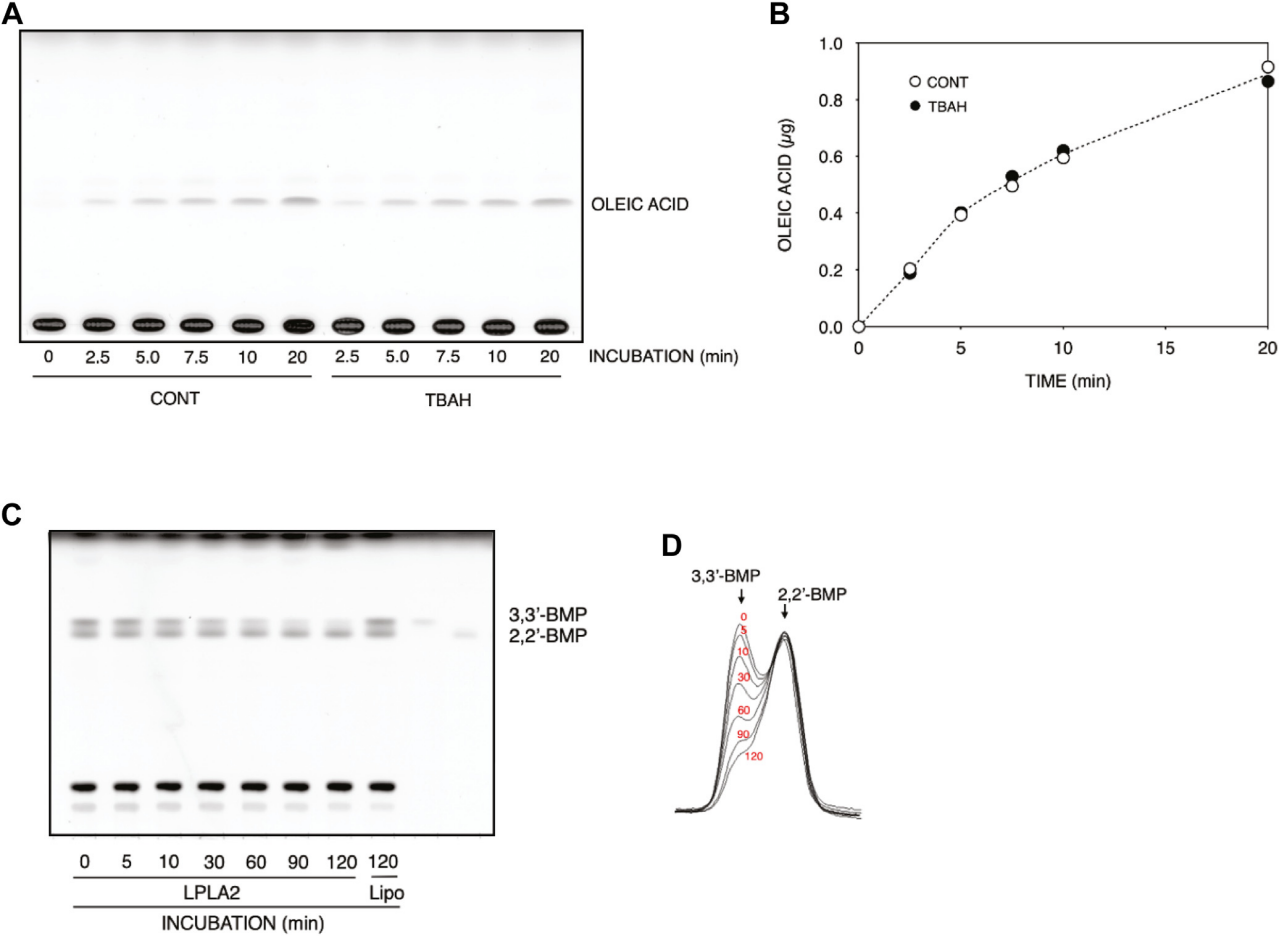
Substrate preference with mixed isomers of BMP. Effect of tetrabutylammonium ion on S,S-(3,3′,C_18:1_)-BMP degradation by hLPLA2. The reaction mixture contained 49 mM sodium acetate (pH 4.5), 10 μg/ml BSA, and 70 μM DODPC and 30 μM S,S-(3,3′,C_18:1_)-BMP as liposomes, in the presence of or in the absence of 30 μM tetrabutylammonium hydroxide. The concentration of LPLA2 was 80 ng/ml. The reaction and lipid extraction were carried out as described in Materials and methods. A and B panels show TLC plate and their kinetics curves, respectively. The plate in panel A was developed in a solvent system consisting of C/M/pyridine (99:1:2, v/v). ACONT and TBAH indicate control (without tetrabutylammonium hydroxide) and tetrabutylammonium hydroxide (30 μM tetrabutylammonium hydroxide), respectively. C and D: Specificity of hLPLA2 for S,S-(2,2′,C_18:1_)-BMP and S,S-(3,3′,C_18:1_)-BMP. The reaction mixture contained 49 mM sodium acetate (pH 4.5), 10 μg/ml BSA, and 70 μM DODPC, 15 μM S,S-(2,2′,C_18:1_)-BMP and 15 μM S,S-(3,3′,C_18:1_)-BMP as liposomes. The reaction was initiated by adding hLPLA2 (the final concentration in the reaction mixture: 100 ng/ml), kept at 37°C, and terminated at the time points indicated in panel C as described in Materials and methods. The changes of both BMP peaks obtained by scanning of the plate in panel C are shown in panel D. 2,2′-BMP and 3,3′-BMP indicate S,S-(2,2′,C_18:1_)-BMP and S,S-(3,3′,C_18:1_)-BMP, respectively. Lipo denotes liposomes without LPLA2.

In the present study, the BMP isomers supplied from Avanti were ammonium salts and S,S-(2,2′,C_18:1_)-BMP from Echelon was a tetrabutylammonium salt. Unlike ammonium ion, tetrabutylammonium ion is a cationic amphiphilic ion. To determine whether tetrabutylammonium ions affect BMP degradation by LPLA2 as observed with cationic amphiphilic drugs, the rate of oleic acid release from DODPC/S,S-(3,3′, C_18:1_)-BMP liposomes in the presence or absence of tetrabutylammonium ions was compared (Fig. 5A, B). The concentration of tetrabutylammonium ion in the reaction mixture was prepared to be the same as that contained in the DODPC/S,S-(2,2′, C_18:1_)-BMP liposome assay. Tetrabutylammonium ion had no significant effect on the hydrolysis of S,S-(3,3′, C_18:1_)-BMP by LPLA2. The substrate preference was then assessed by measuring the hydrolysis of the BMP isomers in liposomes with equal molar ratios of S,S-(2,2′, C_18:1_)-BMP and S,S-(3,3′, C_18:1_)-BMP in the presence of LPLA2. In the LPLA2 assay using DODPC/S,S-(2,2′, C_18:1_)-BMP/S,S-(3,3′, C_18:1_)-BMP (the molar ratio: 70:15:15) liposomes, S,S-(3,3′, C_18:1_)-BMP degraded markedly over time, while S,S-(2,2′, C_18:1_)-BMP showed almost no degradation (Fig. 5C, D).

### Effects of amiodarone on DOPG and BMP isomer degradation by LPLA2

**Fig. 6:**
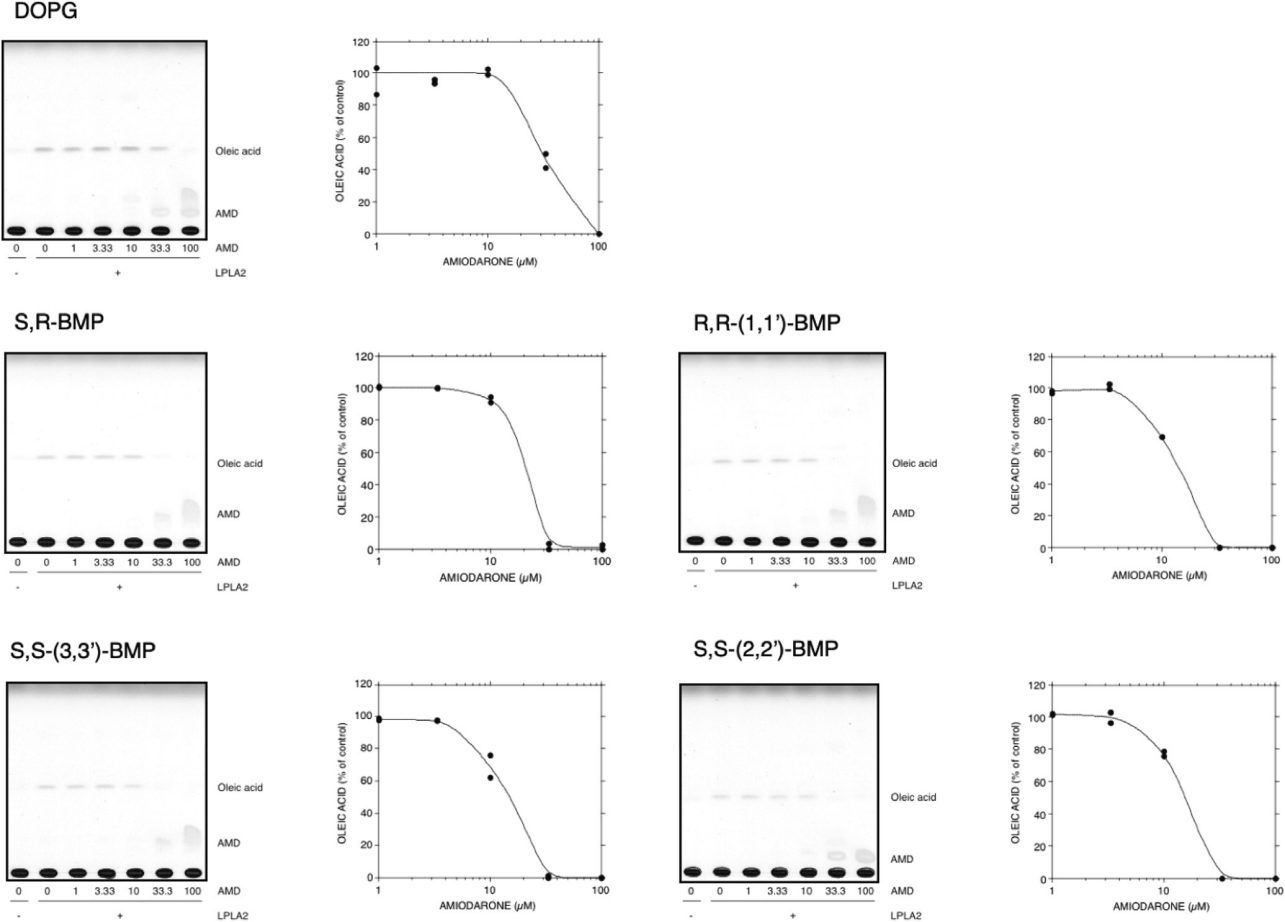
Effect of amiodarone on the degradation of PODG and BMP isomers by LPLA2. LPLA2 was incubated with liposomes consisting of 70 μM DODPC and 30 μM DOPG or 30 μM BMP isomer such as S,R-(3,1′,C_18:1_), R,R-(1,1′,C_18:1_)-, S,S-(3,3′,C_18:1_)- and S,S-(2,2′,C_18:1_)-BMPs in the presence of different concentrations of amiodarone (1, 3.3, 10, 33.3 and 100 μM). LPLA2 activity was determined as described in the Methods section. The incubation times for each isomer were within the linear portion of the reactions as described in supplemental Fig. S1 and were respectively DOPG (2.5 min), R,S-BMP (12 min), R,R-(1,1′-diC_18:1_)-BMP (15 min), S,S-(3,3′-diC_18:1_)-BMP at (8 min), and S,S-(2,2′-diC_18:1_)-BMP (32.5 min). LPLA2activity is expressed as percent of control activity as the average of duplicate assays. Control activity was LPLA2 activity in the absence of amiodarone. The control specific activities measured in duplicate for each substrate were DOPG 77.6 and 80.7 μmol/min/mg protein; S,R-(3,1′,C_18:1_)-BMP 7.84 and 8.18 μmol/min/mg protein; R,R-(1,1′,C_18:1_)-BMP 8.19 and 8.09 μmol/min/mg protein; S,S-(3,3′,C_18:1_)-BMP 13.19 and 13.74 μmol/min/mg protein; and S,S-(2,2′,C_18:1_)-BMP 0.977 and 1.05 μmol/min/mg protein.

To evaluate the effect of CADs on de-acylation of DOPG and BMP isomers by LPLA2, DODPC/DOPG, DODPC/R,S-(3,1′,C_18:1_)-BMP, R,R-(1,1′,C_18:1_)-BMP, S,S-(3,3′ C_18:1_)-BMP or S,S-(2,2′,C_18:1_)-BMP liposomes were pre-incubated with 0, 1, 3.33, 10, 33.3 and 100 μM AMD and then incubated with recombinant hLPLA2. In each case, AMD inhibited LPLA2 activity in a concentration-dependent manner (Fig. 6). The de-acylation activity of LPLA2 for BMPs was almost completely suppressed in the presence of 33.3 μM AMD (Fig. 6). However, about 50% of LPLA2 activity for DOPG was retained in the presence of 33.3 μM AMD. The IC_50_s of AMD for the de-acylation of each BMP isomer and DOPG by LPLA2 were estimated to be less than 20 μM and 30 μM, respectively.

### Effect of LPLA2 on AMD-treated cells

**Fig. 7:**
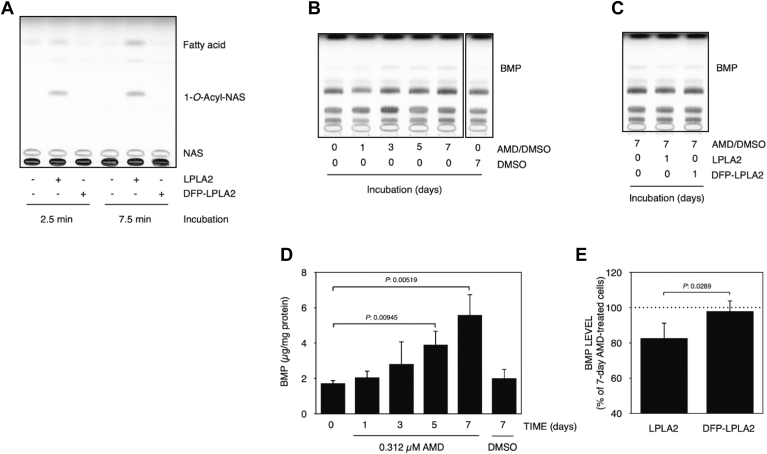
Effects of LPLA2 on cellular BMP accumulation by treatment with AMD. In panel A, recombinant hLPLA2 (85 ng/ml) treated with or without 20 mM DFP was incubated with liposomes (128 μM as phospholipid) consisting of DOPC/sulfatide/NAS (the molar ratio: 10:1:3) for 2.5 and 7.5 min at 37°C at pH 4.5. The reaction products were developed in a solvent system consisting of chloroform/acetic acid (9:1, v/v) (A). DFP-treated LPLA2 was used as inactive hLPLA2 in the cell culture system. See Materials and methods for details. RAW 264.7 cells were exposed with 0.312 μM AMD for 1, 3, 5 and 7 days or with 0.05% DMSO for 7 days. In panel B, cellular phospholipids extracted from AMD-treated or untreated cells were analyzed by TLC as described in Materials and methods. Panel D shows the cellular BMP level and error bars indicate standard deviation (n = 3). Also, the cells after 7-days AMD-treatment were replaced with fresh medium without AMD and incubated with hLPLA2 or inactive hLPLA2 (DFP-LPLA2) for 24 h (C and E). In panel E, the cellular BMP levels treated with these enzymes were compared to the BMP levels of 7-days AMD-treated cells (defined as 100%). Error bars indicate standard deviation (n = 4). The thin layer chromatograms displayed in panels B and C were obtained from the same plate but for clarity are displayed as two separate panels. The last lane in panel B is shown as spliced since it was not adjacent to the fifth lane in the original plate. The fifth lane in panel B and first lane in panel C represent the identical experimental condition and are duplicated in each panel. The raw data for these panels is accessible online at 10.6084/m9.figshare.25768965.

To explore a potential association between AMD-induced LPLA2 inhibition and BMP accumulation, murine macrophage-like cell line RAW 264.7 cells were treated with AMD. In preliminary studies, we confirmed that BMP in wild-type RAW 264.7 cells was detectable by TLC analysis and that RAW 264.7 cells exhibited a higher sensitivity to AMD than common mammalian cells such as MDCK cells. AMD treatment with more than 0.5 μM immediately resulted in cytotoxicity against RAW 264.7 cells. Therefore, to evaluate the effects of AMD in the absence of cytotoxicity, RAW-276.4 cells were exposed to a medium containing 0.312 μM AMD and incubated for 1, 3, 5 and 7 days without changing the media. Cellular BMP levels were increased by treatment with AMD in a time-dependent manner (Fig. 7B, D). The cellular BMP levels at 5 and 7 days after AMD treatment were significantly higher than that of untreated cells (0-days and 7-days treated cells without AMD) (Fig. 7B, D). At the same time, a slight increase in total cell phospholipid levels was observed 3 days after treatment and thereafter (data not shown) although the magnitude of the increase in phospholipid phosphate was smaller compared with BMP. In addition, when 7-days AMD-treated cells were cultured for 24 h in AMD-free fresh medium in the presence of active recombinant LPLA2 or DFP-inactivated recombinant LPLA2, BMP levels in AMD-treated RAW-267.4 cells were significantly reduced by treatment with the active enzyme but not with the inactive enzyme (Fig. 7A, C, E).

### Inhibition of BMP degradation by additional cationic amphiphilic drugs

To evaluate the effect of some CADs on transacylation and deacylation of BMP by LPLA2, DODPC/S,S-(3,3`,C18:1)-BMP/NAS and DODPC/S,S-(3,3`,C18:1)-BMP were pre-incubated with sixteen representative CADs, between 1 and 100 μM for 5 min followed by the addition of hLPLA2. We observed that 14 of previously reported cationic amphiphilic drugs (CADs) inhibit LPLA2 using DOPC for substrate, inhibited LPLA2 using BMP as a substrate with IC50 values less than 63.33 μM. Chloroquine did not inhibit LPLA2 with BMP substrate, as previously observed also when DOPC was used as substrate (Table 1).

**Table 1:** Inhibition of LPLA2-mediated BMP deacylation and transacylation by drugs associated with phospholipidosis

Generic Name	IUPAC Designation	CAS no.	Indication	Transacylase Inhibition 1-*O*-acyl-NAS IC50 μM (DOPC)^g^	Transacylase Inhibition 1-*O*-acyl-NAS IC50 μM (BMP)^g^	Lipase Inhibition IC50 μM (BMP)^g^	ClogP^b^	pKa^c^	Ploemen Value
amiodarone	(2-butyl-1-benzofuran-3-yl)-[4-[2-(diethylamino)ethoxy]-3,5-diiodophenyl]methanone	1951-25-3	antiarrhythmic	8.3	21.6	18.0	7.57	8.48	129
amlodipine	3-O-ethyl 5-O-methyl 2-(2-aminoethoxymethyl)-4-(2-chlorophenyl)-6-methyl-1,4-dihydropyridine-3,5-dicarboxylate	111470-99-6	calcium channel blocker	16.7	18.4	24.0	2.22	9.45	94.2
buclizine	1-[(4-tert-butylphenyl)methyl]-4-[(4-chlorophenyl)-phenylmethyl]piperazine	82-95-1	antihistamine	10.4	6.0	16.0	6.16	8.04	103
carvedilol	1-(9H-carbazol-4-yloxy)-3-[2-(2-methoxyphenoxy)ethylamino]propan-2-ol	610309-89-2	alpha- and beta-adrenergic blocker	13.2	13.6	34.7	3.05	8.74	85.7
chlorpromazine	3-(2-chlorophenothiazin-10-yl)-*N*,*N*-dimethylpropan-1-amine	50-53-3	antipsychotic	9.01	14.1	9.7	5.41	9.3	116
chloroquine	4-N-(7-chloroquinolin-4-yl)-1-N,1-N-diethylpentane-1,4-diamine	54-05-7	Immunosuppressive and antiparasitic	655	ND	ND	4.63	10.23	128
cyclobenzaprine	N,N-dimethyl-3-(2-tricyclo[9.4.0.03,8]pentadeca-1(15),3,5,7,9,11,13-heptaenylidene)propan-1-amine	303-53-7	muscle relaxant	6.24	16.4	13.2	4.73	8.47	94
fosinopril	(2S,4S)-4-cyclohexyl-1-[2-[(2-methyl-1-propanoyloxypropoxy)-(4-phenylbutyl)phosphoryl]acetyl]pyrrolidine-2-carboxylic acid	98048-97-6	angiotensin-converting enzyme inhibitor	0.18	0.91	0.849	5.62	−4.4	31.6
fosinoprilat	(2S,4S)-4-cyclohexyl-1-{2-[hydroxy(4-phenylbutyl) phosphoryl]acetyl}pyrrolidine-2-carboxylic acid	95399-71-6	fosinopril metabolite	5.76	38.6	39.9	3.7	−4.7	35.8
fulvestrant	(7R,8R,9S,13S,14S,17S)-13-methyl-7-[9-(4,4,5,5,5-pentafluoropentylsulfinyl)nonyl]-6,7,8,9,11,12,14,15,16,17-decahydrocyclopenta[a]phenanthrene-3,17-diol	129453-61-8	chemotherapeutic	61.9	20.5	18.5	6.54	−0.88	42.8
lofrepramine	1-(4-chlorophenyl)-2-[3-(5,6-dihydrobenzo[b][1]benzazepin-11-yl)propyl-methylamino]ethanone	23047-25-8	antidepressant	13.3	37.8	41.7	6.11	6.53	80
loratadine	ethyl 4-{13-chloro-4-azatricyclo [9.4.0.03, 8]pentadeca1(11),3(8), 4,6,12,14-hexaen-2-ylidene}piperidine-1-carboxylate	79794-75-5	antihistamine	8.94	52.6	21.8	4.8	4.33	41.8
perhexiline	2-(2,2-dicyclohexylethyl) piperidine	6621-47-2	coronary vasodilator	11.7	6.8	31.1	6.2	10.58	150
profenamine	N,N-diethyl-1-phenothiazin-10-ylpropan-2-amine	522-00-9	anti-dyskinetic	8.7	37.5	40.9	5.75	9.6	125
propranolol	1-(naphthalen-1-yloxy)- 3-[(propan-2- yl)amino] propan-2-ol	525-66-6	beta blocker	51.9	63.3	45.8	3.48	9.67	106
raloxifene	[6-hydroxy-2-(4-hydroxyphenyl)-1-benzothiophen-3-yl]-[4-(2-piperidin-1-ylethoxy)phenyl]methanone	84,449-90-1	estrogen receptor modulator	27.5	30.6	50.6	5.45	8.42	130
SB222200	3-methyl-2-phenyl-N-[(1S)-1-phenylpropyl]quinoline-4-carboxamide	174635-69-9	antihistamine	14.9	31.2	28.7	2.17	9.57	98.2
seproxitine	(3S)-3-phenyl-3-[4-(trifluoromethyl) phenoxy]propan-1-amine	126924-38-7	serotonin reuptake inhibitor	14.6	16.3	20.9	3.8	9.77	110
tamoxifen	(2-[4-[(Z)-1,2-diphenylbut-1-enyl]phenoxy]-N,N-dimethylethanamine	10540-29-1	antiestrogenic	7.7	10.8	20.1	5.93	8.76	112

The generic name, International Union of Pure and Applied Chemistry (IUPAC) designation, chemical abstracts registry (CAS) number, and clinical indication are provided. Chemical properties, including ClogP and pKa (basic) were obtained from the Pubchem and chEMBL database of bioactive molecules. LPLA2 IC_50_ denotes the concentration at which 50% of the LPLA2 dependent 1-O-acyl N-acetylsphingosine synthase activity is observed (as previously published, b) transacylase (DODPC/BMP/NAS liposomes in molar ratio1:2.3:1.3, c) lipase activity DODPC/S,S-(3,3′)-BMP in the molar ratio of 2.3:1, ClogP, a measure of partitioning between octanol and water, predictive of transport independent distribution across cell membranes, pKa (basic) is a determinant of the protonation of an amine at lysosomal pH. ClogP and pKa (basic) were employed by Ploemen and colleagues to generate an in silico model that is predictive of phospholipidosis and used to calculate the Ploemen value for each compound. By convention, negative pKa values were assigned a value of 0 for calculation of the Ploemen number and would be predicted negative based on pKa values of less than 8 or 6 for the Ploemen and modified Ploemen models respectively. The graphed data from which the IC50 values were derived can be accessed at 10.6084/m9.figshare.25768965.

## Discussion

We report two significant findings in this paper. First, BMP isomers are substrates for LPLA2, a finding originally suggested in studies of alveolar macrophages from *Lpla2*−/− mice (7). This conclusion is supported by the demonstrable release of fatty acid and by the formation of 1-*O*-acyl-*N*-acetylsphingosine. Although other phospholipase A2s are known to act as transacylases, the recognition of *N-*acetyl-sphingosine as an acceptor and the formation of 1-*O*-acyl-C2 ceramide by LPLA2 is a unique property of lysosomal phospholipase A2. Second, several compounds associated with drug-induced phospholipidosis inhibit both the lipase and transacylase activities of LPLA2 in the presence of BMP substrate. This latter finding provides a potential explanation for the observed increase in plasma BMP levels in animal models of phospholipidosis. BMP is currently accepted as a biomarker for this form of drug toxicity (15).

### Degradation of BMPs by LPLA2

We have previously observed that LPLA2 shows a broad positional specificity for acyl groups of glycerophospholipids (16) and higher affinity and hydrolase activity toward acidic glycerophospholipids, including phosphatidylglycerol (PG) and cardiolipin, compared to neutral glycerophospholipids (17). PG, a precursor of BMP, is among the best substrates for LPLA2 (17). In contrast, S,S-(2,2′-diacyl)-BMPs are thought to be as naturally occurring forms, which are isomers of PG and are located in acidic compartments, and to be resistant to phospholipases and lipases due to their unusual stereochemical configuration (18).

DODPC is resistant to LPLA2 activity. Using DODPC-based BMP liposomes, we confirmed that LPLA2 catalyzes the deacylation as well as transacylation of S,S-(2,2′,C_18:1_)-BMP under acidic conditions, although the fatty acid release by LPLA2 for S,S-(2,2′,C_18:1_)-BMP is 60–70 times lower than that for DOPG (Figs. 1 and 2). However, the activity observed for S,S-(2,2′,C_18:1_)-BMP as a substrate is comparable to the activity observed previously in the DODPC/1,2-dioleoylphosphatidylcholine liposomes (17). This suggests that S,S-(2,2′,C_18:1_)-BMP can be recognized as a substrate for LPLA2, like other glycerophospholipids, under the acidic conditions found in the lysosome. LPLA2 also degraded other isomers of S,S-(2,2′,C_18:1_)-BMP. Comparing the substrate specificity of LPLA2 based on initial velocity, LPLA2 showed 8-, 8-, and 13-fold higher activity for R,S-(3,1′,C_18:1_)-, R,R-(1,1′,C_18:1_)- and S,S-(3,3′,C_18:1_)-BMPs, respectively, than for S,S-(2,2′,C_18:1_)-BMP (Fig. 3). Differences in substrate specificity of LPLA2 for BMP isomers were shown more clearly by using the DODPC liposomes mixed with S,S-(3,3′,C_18:1_)-BMP and S,S-(2,2′,C_18:1_)-BMP (Fig. 5C, D).

S,S-(2,2′,C_18:1_)-BMP is a symmetrical diacylglycerol phospholipid with a unique stereo-configuration, *sn*-1-glycerophosphate-*sn*-1′-glycerol, and is a poor substrate for other phospholipase A2s. S,S-(3,3′,C_18:1_)-BMP is formed by the acyl migration of S,S-(2,2′,C_18:1_)-BMP, is thermodynamically more stable than S,S-(2,2′,C_18:1_)-BMP, and is a better substrate for LPLA2 than the other BMPs. A study comparing the structure and dynamics of the 2,2′ and 3,3′ isoforms suggested that the latter form is more flexible and has a greater tendency to adopt parallel chains perhaps accounting for its improved substrate recognition by LPLA2 (19).

Among the studied BMP isomers, S,S-(2,2′)-BMP is the only isomer having the acyl groups conjugated with the hydroxyl groups at the *sn*-2 and *sn*-2′ chiral carbon atoms. The carbon atoms conjugated with the acyl groups of other BMP isomers are not chiral. In general, the acyl group attached to the chiral carbon atom in the R configuration of diacylglycerophospholipids is a better leaving group than the acyl group in the S configuration in the phospholipase A2 reaction (1). This may explain the lower activity of LPLA2 for S,S-(2,2′,C_18:1_)-BMP compared to other dioleoyl-BMP isomers. In addition, studies on the isomerization of 1-lyso-2-acyl-phosphatidylcholine report that a substantial amount of 1-lyso-2-acyl-PC in plasma is isomerized to 1-acyl-2-lyso-PC under neutral conditions but not at pH 4.5 (20, 21). Therefore, an increase in luminal pH induced by lysosomal membrane permeabilization or lysosomal rupture may possibly promote the conversion of S,S-(2,2′)-BMP to S,S’-(3,3′)-BMP.

Damaged lysosomes are removed by a type of selective macroautophagy, termed lysophagy (22). S,S’-(3,3′)-BMPs produced by conversion from S,S-(2,2′)-BMPs could be more efficiently degraded by LPLA2 in the acidic compartment of the lysosome/autophagosome system and this 2,2′ to 3,3′-acyl migration could play a key role in promoting lysosome clearance by lysophagy.

### Effects of amiodarone on accumulation and degradation of cellular BMP

Our in vitro study using DODPC/DOPG or BMP liposomes and different concentrations of AMD showed that hLPLA2 activities for individual isomers were inhibited by AMD in a dose-dependent manner (Fig. 6). These results indicate that the inhibition of LPLA2 activity on BMPs by AMD similarly occurs via a mechanism proposed for other diacylgycerophospholipids, in which LPLA2 activity on phospholipids is primarily regulated through electrostatic interaction between the enzyme and phospholipid membrane (13, 23). In this model, AMD interacts with the membrane, cancels the negative charge in the membrane, and reduces an electrostatic interaction between positively charged LPLA2 and phospholipid membrane under acidic conditions. An important exception to this model was chloroquine, a known cause of phospholipidosis. No inhibition of LPA2 was observed in the presence of this agent suggesting that it acts through an alternative mechanism. One possibility is that chloroquine raises lysosomal pH to a level that is less favorable for LPLA2 activity.

BMPs were more sensitive to inhibition of catabolism by AMD than was PG (Fig. 6). Both PG and BMPs are acidic phospholipids and tend to interact with lipophilic cationic compounds such as CADs. The molecular shapes of BMPs and PG are inverted cones and cylinders, respectively, which affect their mobility in the lipid membrane. It is possible that BMP molecules interact with AMD more efficiently than PG to reduce the negative charge of the liposome membrane, resulting in the difference in IC_50_ between PG and BMP regarding the AMD inhibitory effect on LPLA2 activity.

Ideally, formal kinetic analyses probing the specificities of LPLA2 toward the various BMP isomers might inform this study. As a practical matter, due to the limited availability of hLPLA2 coupled with the relative insensitivity of the TLC assay system, we were unable to consider such analyses. However, it is well established that such studies are “plagued” by the physical state of the lipid substrates for a particular enzyme which varies regarding their head groups and fatty acyl chains (24). Unique to this study are the structural variations of the 2,2′- and 3,3′-BMP species that are secondary to their unique isomerization where their differences in shape have likely effects on membrane curvature including in the liposomes used in the LPLA2 assay. Therefore, the interpretation of kinetic data that relies on assays that only vary with the presence of one or another isomer is problematic. While this challenge could potentially be addressed by the use of a mixed micelle assay system, we observed in our original work on the purification and characterization of lysosomal phospholipase A2, that detergents including Triton X-100 significantly inhibited the activity of the enzyme in a concentration-dependent manner (25). This finding has discouraged us from pursuing mixed micelle systems for the enzyme assay and more generally in pursuing kinetic analyses which are vulnerable to misinterpretation.

Other enzymes have been reported to deacylated BMP including phospholipase A2/lipase, a member of carboxylesterase of the family (26), pancreatic lipase-related protein 2 (PLRP2) (27), and the α/β hydrolase domain (ABDH) enzymes, ABDH6 (28) and ABDH12 (29). Among these lipases, ABDH6 originally reported as a monoacylglycerol hydrolase, is specifically located on the cytosolic surface of the lysosome limiting membrane. Unlike LPLA2, the pH optimum of the ABHD6 acylase activity for BPM isomers is 8 in the presence of Chaps detergent, and the values of *Km* and *Vm* for S,S-(2,2′)-BMP of ABDH6 are 0.98 mM and 5.8 μmol/min/mg protein, respectively (28). By comparison, the specific activities of LPLA2 for S,R-(3, 1′,C_18:1_)-BMP, R,R-(1,1′)-BMP, S,S-(3,3′)-BMP and S,S-(2,2′)-BMP are 8.35 ± 0.44, 8.30 ± 0.136, 13.4 ± 0.559 and 1.03 ± 0.034 μmol/min/mg protein, respectively (Fig. 4).

The assay conditions for ABDH6 differ from those in this study where the lipase and transacylase reactions are run under acidic conditions. However, the concentrations of DOPG and BMPs (30–40 μM) used in our assay system are much lower than the *Km* value for S,S-BMP of ABHD6. Thus, LPLA2 may possess a much greater activity toward BMPs under optimum conditions than the specific activities of LPLA2 shown above. In addition, ABDH6 deficiency in mice treated with AMD promotes BMP release into the circulation but is not associated with hepatic BMP accumulation and with the formation of the multilamellar bodies characteristic of phospholipidosis (30). Based on the cellular location and enzymatic properties of AHBD6 and LPLA2, and the AMD-responsive phenotype of AHBD6 or LPLA2 deficient mice, it is possible that both enzymes have a role in the catabolism of BMP, in which ABDH6 and LPLA2 act at the cytosolic site and the luminal sides of the lysosome, respectively.

## Data availability

All data is available in the main text, supplementary figure, or online at 10.6084/m9.figshare.25768965.

## Supplemental data

This article contains supplemental data.

## Conflict of interests

The authors declare that they have no known competing financial interests or personal relationships that could have appeared to influence the work reported in this paper. The author is an Editorial Board Member/Editor-in-Chief/Associate Editor/Guest Editor for *The Journal of Lipid Research* and was not involved in the editorial review or the decision to publish this article.
